# Examining the effect of smoking on suicidal ideation and attempts: triangulation of epidemiological approaches

**DOI:** 10.1192/bjp.2020.68

**Published:** 2020-12

**Authors:** Ruth Harrison, Marcus R. Munafò, George Davey Smith, Robyn E. Wootton

**Affiliations:** 1 Avon & Wiltshire Mental Health Partnership NHS Trust; and Severn Postgraduate Medical Education School of Psychiatry, Health Education England, UK; 2 School of Psychological Science, University of Bristol; MRC Integrative Epidemiology Unit, University of Bristol; NIHR Biomedical Research Centre, University Hospitals Bristol NHS Foundation Trust, University of Bristol; and UK Centre for Tobacco and Alcohol Studies, University of Bristol, UK; 3 MRC Integrative Epidemiology Unit, University of Bristol; and Department of Population Health Sciences, Bristol Medical School, University of Bristol, UK; 4 School of Psychological Science, University of Bristol; MRC Integrative Epidemiology Unit, University of Bristol; and NIHR Biomedical Research Centre, University Hospitals Bristol NHS Foundation Trust, University of Bristol, UK

**Keywords:** Suicide, smoking, epidemiology, tobacco, Mendelian randomisation

## Abstract

**Background:**

Previous literature has demonstrated a strong association between cigarette smoking, suicidal ideation and suicide attempts. This association has not previously been examined in a causal inference framework and could have important implications for suicide prevention strategies.

**Aims:**

We aimed to examine the evidence for an association between smoking behaviours (initiation, smoking status, heaviness, lifetime smoking) and suicidal thoughts or attempts by triangulating across observational and Mendelian randomisation analyses.

**Method:**

First, in the UK Biobank, we calculated observed associations between smoking behaviours and suicidal thoughts or attempts. Second, we used Mendelian randomisation to explore the relationship between smoking and suicide attempts and ideation, using genetic variants as instruments to reduce bias from residual confounding and reverse causation.

**Results:**

Our observational analysis showed a relationship between smoking behaviour, suicidal ideation and attempts, particularly between smoking initiation and suicide attempts (odds ratio, 2.07; 95% CI 1.91–2.26; *P* < 0.001). The Mendelian randomisation analysis and single-nucleotide polymorphism analysis, however, did not support this (odds ratio for lifetime smoking on suicidal ideation, 0.050; 95% CI −0.027 to 0.127; odds ratio on suicide attempts, 0.053; 95% CI, −0.003 to 0.110). Despite past literature showing a positive dose-response relationship, our results showed no clear evidence for a causal effect of smoking on suicidal ideation or attempts.

**Conclusions:**

This was the first Mendelian randomisation study to explore the effect of smoking on suicidal ideation and attempts. Our results suggest that, despite observed associations, there is no clear evidence for a causal effect.

There are more than 800 000 deaths from suicide each year,^1^ and for each death, there are 10–40 unsuccessful attempts.^2^ The World Health Organization has recognised this significant public health problem and the need for comprehensive suicide prevention strategies; however, at present, there is limited evidence of sustained reductions in suicides rates.^1^ Observational studies, including case-control, cohort and cross-sectional designs, using population data^3–13^ and clinical samples,^14^ have demonstrated a strong association between current cigarette smoking and suicide-related behaviours characterised as ideation, plans, attempts and suicide-related death. The association remains in three meta-analyses of observational studies.^15–17^ These associations have been shown to have positive dose-response relationships^7,11,18^ that remain after adjustment for potential confounding variables such as psychiatric symptoms,^5^ familial risk factors,^12^ socioeconomic characteristics^6^ and alcohol consumption.^19^ Smoking interventions such as imposing cigarette taxes and smoke-free air policies are reported to be protective against suicide-related outcomes.^8^

## Possible causal pathways

A number of psychopathological and physiological hypotheses have been explored to determine whether this association is causal. A number of possible biological pathways have been explored,^7^ including evidence that smoking lowers the levels of serotonin^14^ and monoamine oxidase A.^20^ Reduced levels of these neurotransmitters are related to depressive episodes and low levels of serotonin are also linked to increased impulsivity.^14^ Nicotine has been found to act as a potent activator of the hypothalamic-pituitary-adrenal axis and this has been linked to suicidal behaviour.^21^ There is evidence to suggest that chronic cigarette smoking has long-term neurocognitive effects, which lead to increased impulsivity and difficulties with decision-making owing to impairments in cognitive flexibility.^7,17^ Alternatively, smoking causes physical health problems, including pain, sleep difficulties, chronic obstructive pulmonary disorder, cardiovascular disease and cancer, which in turn could lead to suicide-related behaviours.^18^

However, even after controlling for specific confounding variables, residual confounding could still be biasing estimates;^6^ for example, as a result of social deprivation, lower levels of education and higher levels of impulsivity.^18,22^ A negative control analysis demonstrated that smoking behaviour predicted the risk of being murdered to the same extent as the risk of suicide, which supports the notion of residual confounding.^6^ Another hypothesis is of reverse causation, namely that individuals with suicidal thoughts may be more likely to smoke, and in addition their suicidality could lead to reduced motivation for smoking cessation. There are several possible explanations for reverse causation, with the most common hypothesis being that individuals use nicotine to self-medicate.^22^ Alternatively, individuals with mental illness might misattribute the relief of nicotine withdrawal as relieving psychological stress, and therefore continue to smoke.^23^

## Applying causal inference techniques

A clear understanding of the relationship between smoking and suicide remains to be established. A recent study demonstrated a causal link between smoking and risk of depression, using a Mendelian randomisation approach.^24^ Mendelian randomisation can be implemented as a type of instrumental variable analysis in which genetic variants known to be associated with the exposure (smoking) are used as an instrument to test for an effect on the outcome (suicidal ideation and suicide attempts). In this study, we apply Mendelian randomisation techniques, using genetic variants identified in genome-wide association studies (GWAS), to the relationship between smoking and suicide attempts and ideation. Previous observational research in this area could be biased by residual confounding and reverse causation, and Mendelian randomisation is one way to reduce bias from these sources.^25^ In our analysis, we looked at smoking initiation, smoking heaviness and lifetime smoking, using a genetic instrument that takes into account smoking status, duration, heaviness and cessation.^24^

## Methods

### Observational analysis

#### Sample

The UK Biobank is a research resource of health data collected on over 500 000 individuals from study centres located across the UK. Recruitment occurred between 2006 and 2010. Participants were aged from 39 to 70 years at recruitment (mean 56.91 years, s.d. 7.99 years) and 54% of the sample were female. Overall, 30% of the sample had ever smoked (8% current smokers and 22% former smokers). Further information is available elsewhere (https://www.ukbiobank.ac.uk/).

#### Measure of suicidal ideation

In the UK Biobank, participants were asked as part of a questionnaire on depressive symptoms ‘Over the last 2 weeks, how often have you been bothered by any of the following problems? Thoughts that you would be better off dead or of hurting yourself in some way’ (field 20513). Participants could respond depending on frequency of thoughts either ‘not at all’, ‘several days’, ‘more than half the days’ or ‘nearly every day’. We recoded these into a binary variable in which those who responded ‘not at all’ were coded as 0 and everyone else was coded as 1. Individuals who responded ‘prefer not to answer’ were coded as missing.

#### Measure of attempted suicide

In the UK Biobank, participants were first asked ‘Have you deliberately harmed yourself, whether or not you meant to end your life?’ (field 20480). If they responded yes, then they were asked ‘Have you harmed yourself with the intention to end your life?’ (field 20483). We used both of these measures to derive one binary measure of suicide attempt in which participants were given a score of 0 if they responded negatively to either question, and a score of 1 if they responded affirmatively to both questions. Individuals who responded ‘prefer not to answer’ were coded as missing.

#### Measure of smoking behaviours

Participants in the UK Biobank self-reported their smoking status (field 20116). All ever smokers were asked to report their average number of cigarettes per day (fields 3456 and 2887). Participants who responded ‘do not know’ or ‘prefer not to answer’ were coded as missing.

#### Statistical analysis

After restricting to individuals of European ancestry with genetic data available (to make this analysis comparable with subsequent analyses), 337 053 individuals remained. We looked at the effect of four smoking behaviours on suicidal ideation and attempts. These were smoking status (ever versus never), smoking status (current versus former within ever smokers), cigarettes per day (within ever smokers) and lifetime smoking score. The latter is a combination of smoking duration, smoking cessation and smoking heaviness described in detail elsewhere.^24^ The effect of each of these smoking behaviours on suicidal ideation and attempts was estimated by logistic regression, controlling for age, gender and socioeconomic position. All analyses were conducted with R software, version 3.5.1, for Mac OS X (https://cran.r-project.org/bin/macosx/).^26^

### Mendelian randomisation analysis with summary-level data

#### Smoking instrument

This Mendelian randomisation approach requires GWAS publicly released summary data from two independent samples. The GWAS of suicide attempt was conducted in the UK Biobank, therefore we were unable to use the lifetime smoking instrument because it was also constructed with the UK Biobank.^24^ Instead, we used the smoking initiation (ever versus never) GWAS conducted by the GWAS and Sequencing Consortium of Alcohol and Nicotine use (GSCAN), taking beta values from the GWAS with UK Biobank removed.^27^ Because of data-sharing restrictions, 23andMe were also removed from the summary data used. GSCAN identified 378 conditionally independent, genome-wide significant single nucleotide polymorphisms (SNPs) associated with smoking initiation, explaining 4% of the phenotypic variance.^27^

#### Suicide attempt GWAS

The GWAS of suicide attempts was conducted in the UK Biobank, using the question and method outlined above, with 337 199 participants of which 2433 were cases.^28^ The authors did not identify any genome-wide significant SNPs, but the summary statistics can be used as an outcome sample in summary-level Mendelian randomisation.

#### Statistical analysis

All analysis was conducted using the TwoSampleMR package^29^ in R version 3.5.1, for Mac OS X (https://cran.r-project.org/bin/macosx/).^26^ We used five different Mendelian randomisation methods: inverse-variance weighted, Mendelian randomisation (MR) Egger,^30^ weighted median,^31^ weighted mode^32^ and MR robust adjusted profile score (RAPS) (MR RAPS).^33^ Each method makes different assumptions and therefore a consistent effect across multiple methods strengthens causal evidence.^34^ If an SNP was unavailable in the outcome GWAS summary statistics, then proxy SNPs were searched for with a minimum linkage disequilibrium *r*^2^ = 0.8 and palindromic SNPs were aligned if minor allele frequency <0.3. We also performed Rucker's *Q*-test of heterogeneity and the MR Egger intercept test to estimate potential directional pleiotropy.^30^ Finally, we performed Steiger filtering, which can indicate possible reverse causation.^35^

### Mendelian randomisation analysis with individual-level data

#### Genotyping

UK Biobank participants provided blood samples at their initial assessment centre. Genotyping was performed with the Affymetrix UK BiLEVE Axiom array for 49 979 participants and the Affymetrix UK Biobank Axiom array for 438 398 participants. The two arrays share 95% coverage, but chip is adjusted for in all analyses because the UK BiLEVE sample is overrepresented for smokers. Imputation and initial quality control steps were performed by the Wellcome Trust Centre for Human Genetics, resulting in over 90 million variants.^36^

Individuals were excluded if there were gender mismatches between reported and chromosomal gender or aneuploidy (*N* = 814). Medical Research Council Integrative Epidemiology Unit filtering restricted the sample to individuals of European ancestry based on the first four principal components of population structure.^37^ After excluding individuals who had withdrawn consent, 463 033 participants remained.^37^ We restricted our analysis to autosomes only, and used stringent filtering thresholds for SNPs of minor allele frequency <0.01 and info >0.8.

#### Conducting the GWAS

Participants from the UK Biobank were randomly allocated to one of two split halves of the genetic data. We then generated lifetime smoking scores in sample one of these two samples, and ran a GWAS with the UK Biobank pipeline,^38^ following the exact method as described elsewhere.^24^

#### Genetic instrument

From the GWAS, genome-wide significant variants (*P* < 5 × 10^−8^) were clumped for independence at 10 000 kb and *r*^2^ < 0.001.

#### Instrument validation

We tested the validity of this instrument by creating a polygenic score from these variants in the second sample from the UK Biobank. This was done with Plink version 1.9, in Linux (https://www.cog-genomics.org/plink/1.9/), and by weighting each allele by the effect size identified in the GWAS of sample one.^39^ This therefore provides an independent replication sample to check how much of the variance is explained in lifetime smoking. If this significantly predicts lifetime smoking in the independent second sample, then this can be used as an instrument in the individual-level Mendelian randomisation analysis.

#### Statistical analysis

We conducted individual-level Mendelian randomisation with instrumental variable regressions run in R version 3.5.1, for Mac OS X (https://cran.r-project.org/bin/macosx/),^26^ with the ivreg command from the AER package. The instrument was the polygenic score from the GWAS in sample one. We controlled for age, gender and ten principal components of population structure in all analyses apart from when we ran the analysis separately in males and females (removing gender as a covariate).

### Single SNP analysis

#### Statistical analysis

Best guess genotypes at the SNP rs1051730 were extracted with Plink version 1.9, in Linux (https://www.cog-genomics.org/plink/1.9/),^39^ in the UK Biobank full sample described above. This SNP in the gene cluster *CHRNA5-A3-B4* is known to be strongly associated with heaviness of smoking.^40–43^ The variant affects the structure of neuronal nicotinic acetylcholine receptors (nAChR) such that nicotine is less able to bind. Individuals with risk alleles that produce nAChR deficiency are able to withstand higher nicotine levels because they are less sensitive to its aversive effects.^44^ As a result, individuals smoke on average one more cigarette per day per risk allele.^43^ We used logistic regression to test whether the number of effect alleles (A) of this SNP were associated with risk of suicidal ideation and suicide attempts, again using the measures described above. We controlled for age and gender in all analyses. The logistic regressions were run in each category of smoking status separately (ever, current, former and never smokers). A causal effect of smoking on suicidal ideation or attempts would be characterised by an effect of rs1051730 in all categories of smoking status apart from never smoking, which provides a negative control.

### Sensitivity analysis

#### Smoking and impulsivity

We wanted to ensure that any effects of smoking on suicide attempts were not the result of confounding from personality factors (e.g. impulsivity and risk-taking) rather than direct effects of smoking. Unfortunately, multivariable Mendelian randomisation was not possible because of sample overlap in our risk-taking and suicide attempt GWAS, which both used the UK Biobank. Therefore, we conducted a follow-up analysis, using bi-directional Mendelian randomisation of smoking initiation on risk-taking behaviour, with summary-level data. As the instrument for smoking initiation, we used the 378 SNPs from the GSCAN consortium GWAS and effect sizes with UK Biobank removed.^27^ For risk-taking behaviour, we used the Social Science Genetic Association Consortium GWAS meta-analysed across multiple cohorts of European ancestry,^45^ which identified 124 SNPs associated with risk tolerance. These analyses followed the method described above for summary-level data.

### Ethical approval

UK Biobank has received ethics approval from the UK National Health Service's National Research Ethics Service (reference 11/NW/0382) and this work is part of approved project 9142.

## Results

### Observational analysis

Of the 109 649 individuals who had responded to the question of suicidal ideation, 4515 (4%) had had suicidal thoughts. Of the 110 035 individuals who had responded to the questions of suicide attempts and self-harm, 2405 (2%) had ever attempted suicide. Using logistic regression, every smoking behaviour increased odds of suicidal attempts and ideation, with the greatest effect being of initiating smoking on odds of attempting suicide (Table 1).

**Table 1.** The observed association of smoking behaviour on suicidal ideation and suicide attempts controlling for age, gender and socioeconomic position

	Suicidal ideation (yes/no)			Suicide attempt (yes/no)		
Smoking	*N*	Odds ratio (95% CI)	*P*-value	*N*	Odds ratio (95% CI)	*P*-value
Ever versus never	109 301	1.39 (1.31–1.48)	<0.001	109 688	2.07 (1.91–2.26)	<0.001
Current versus former	45 825	1.54 (1.39–1.70)	<0.001	46 011	1.54 (1.36–1.75)	<0.001
Cigarettes per day	29 230	1.02 (1.01–1.02)	<0.001	29 342	1.02 (1.02–1.03)	<0.001
Lifetime smoking	109 301	1.42 (1.36–1.48)	<0.001	109 688	1.80 (1.71–1.89)	<0.001

### Mendelian randomisation analysis with summary-level data

Of the 378 conditionally independent SNPs associated with smoking initiation identified by the GSCAN consortium,^27^ 321 were available in the GWAS summary data for suicide attempt.^28^ We first performed the Rucker's *Q*-test of heterogeneity, which did not provide evidence for heterogeneity (Supplementary Table 1 available at https://doi.org/10.1192/bjp.2020.68). MR Egger analysis could not be conducted because the regression dilution 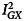 was below 0.6 (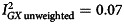). All of the other four Mendelian randomisation methods showed the same direction of effect, with smoking initiation increasing the odds of attempting suicide (Table 2). The strongest evidence was from the inverse-variance weighted and MR RAPS methods. The evidence was weaker for the weighted median and weighted mode approaches, which make different assumptions about the nature of pleiotropy. However, the MR Egger intercept and Rucker's *Q*-tests showed no clear evidence of bias from directional pleiotropy (Supplementary Table 2). Steiger filtering estimated that over half of the genetic instruments explained more variance in the outcome than the exposure, suggesting that there might be reverse causation (Supplementary Table 3).

**Table 2.** Mendelian randomisation analyses using summary-level data of smoking initiation on risk of suicide attempt

Method	SNPs	Odds ratio (95% CI)	*P*-value
Inverse-variance weighted	321	2.42 (1.53–3.83)	<0.001
Weighted median	321	1.73 (0.85–3.50)	0.13
Weighted mode	321	1.93 (0.24–15.28)	0.53
Mendelian randomisation RAPS	321	2.86 (1.64–4.98)	<0.001

SNP, single nucleotide polymorphism; RAPS, robust adjusted profile score.

### Mendelian randomisation analysis with individual-level data

To conduct a Mendelian randomisation of lifetime smoking behaviour on risk of suicidal ideation and suicide attempt with individual-level data, we first had to conduct a GWAS of suicide attempt in half of the UK Biobank sample, using a random split. We identified 19 independent, genome-wide significant SNPs associated with lifetime smoking score. These were then extracted from the second half of the UK Biobank sample (with no sample overlap) and weighted by the effect size to create a polygenic score for each individual. The second half of the UK Biobank sample is 54% female, with mean age 56.88 years (s.d. 8.00 years). Mean lifetime smoking score in the second half of the sample was 0.342 (s.d. 0.679). A total of 4% of individuals had experienced suicidal thoughts (4% of females and 4% of males), and 2% of individuals had caused themselves harm with the aim to end their life (3% of females and 2% of males). We tested the association of lifetime smoking score and polygenic risk score on the baseline confounders of gender, age, socioeconomic position, alcohol consumption and educational attainment (Supplementary Table 4). For all confounders, the association was attenuated for the polygenic risk score compared with the observed association.

We validated that the score predicts smoking behaviour by conducting a linear regression of polygenic score on lifetime smoking behaviour in the second half of the UK Biobank. It explained 0.171% (*P* < 0.001) of the variance in lifetime smoking behaviour. Finally, we conducted the individual-level Mendelian randomisation analysis of lifetime smoking polygenic risk score on suicidal ideation and suicide attempt, controlling for age, gender and ten principal components of population structure (Table 3). There was no clear evidence for an effect of lifetime smoking on suicidal ideation or suicide attempt, but a trend toward increased risk in both analyses (Table 3).

**Table 3.** Mendelian randomisation analysis of lifetime smoking on suicidal ideation and attempt using individual-level data

	Suicidal ideation			Suicide attempt		
	*N*	Beta (95% CI)	*P*-value	*N*	Beta (95% CI)	*P*-value
All	54 289	0.050 (−0.027 to 0.127)	0.21	54 500	0.053 (−0.003 to 0.110)	0.06
Females	30 480	0.069 (−0.047 to 0.185)	0.24	30 590	0.046 (−0.047 to 0.140)	0.33
Males	23 797	0.028 (−0.073 to 0.130)	0.59	23 898	0.060 (−0.004 to 0.124)	0.07

### Single SNP analysis

**Fig. 1:**
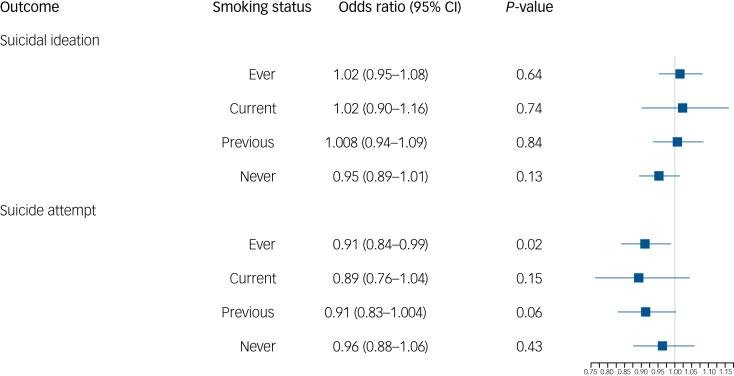
Logistic regression results of genotype at rs1051730 on odds of suicidal ideation and suicide attempt by smoking status.

Finally, we extracted values for the SNP rs1051730 (A/G) from individuals in the UK Biobank. We first confirmed that an increased number of effect alleles (A) were associated with increased smoking behaviour (Supplementary Table 5), where we observed the anticipated increase of approximately one cigarette more per day per allele within ever smokers. We also showed that genotype at rs1051730 is not associated with smoking status or the baseline confounders of gender or alcohol consumption (Supplementary Table 5). However, there was some evidence to suggest that genotype at rs1051730 was associated with educational attainment and age (Supplementary Table 5). There was no clear evidence for an effect of rs1051730 genotype on suicide attempts or ideation controlling for age and gender (Fig. 1). There was weak evidence to suggest that the number of rs1051730 effect alleles might reduce risk of suicide attempts, with no effect in the never smokers and a small protective effect in the ever smokers (Fig. 1). If smoking is increasing risk of suicidal behaviour, we would still expect to see no association in never smokers and the opposite effect within ever smokers.

### Smoking and impulsivity sensitivity analysis

We only saw evidence for smoking as a risk factor for suicide attempts when the instrument was smoking initiation. One trait associated with both smoking initiation and suicide attempts is impulsivity. Therefore, we conducted a bi-directional Mendelian randomisation of smoking initiation and risk-taking, using summary-level data, which have previously been shown to be genetically correlated.^45^ There was strong evidence of a bi-directional causal relationship between smoking initiation and risk-taking behaviour, suggesting that the smoking initiation SNPs might be capturing an underlying impulsivity phenotype (Supplementary Table 6), which could explain why we only observed effects of smoking initiation on suicidal ideation and attempts and not for the other smoking phenotypes. Rucker's *Q*-tests showed some evidence of heterogeneity (Supplementary Table 1) and the MR Egger intercept showed weak evidence of bias by directional pleiotropy (Supplementary Table 2).

## Discussion

The relationship between smoking and suicide-related behaviour is not clearly understood and has important clinical implications.^1,2^ In this study, our observational analysis replicated previous observed associations between smoking behaviour and suicidal ideation and attempts, particularly between smoking initiation and suicide attempts. We went on to explore this association with a Mendelian randomisation approach to provide evidence about whether or not the association is causal. Overall, the Mendelian randomisation analyses, including the single SNP analysis, did not support a causal interpretation. Therefore, despite past literature showing a positive dose-response relationship, our results do not support a causal effect of smoking on suicide attempts or ideation. Our findings support those of a negative control analysis demonstrating that smoking behaviour predicted murder as strongly as it did suicide.^6^

Our Mendelian randomisation results show evidence of a relationship between smoking initiation and suicidal ideation, but little evidence of an effect of lifetime smoking on suicidal ideation. Taken together with weak evidence for a protective effect in the single SNP analysis, these triangulated results overall suggest that there is no clear evidence for an effect of smoking on increased risk of suicidal ideation or attempts.

### The potential role of impulsivity

The only evidence for smoking as a risk factor was when smoking initiation was the genetic instrument. However, our follow-up analyses suggest this could be owing to the instrument capturing underlying impulsivity. Smoking initiation is a complicated instrument with both behavioural and biological components. This behavioural component is likely to be related to impulsivity in part. This is supported by high genetic correlations between the two phenotypes.^45^ As there is a known correlation between impulsivity and suicidal behaviours, we were mindful of this association. Therefore, to examine the effect of impulsivity on our results, we undertook a bi-directional Mendelian randomisation of smoking initiation and risk-taking. We showed strong evidence of a bi-directional causal relationship between smoking initiation and risk-taking behaviour, suggesting that our results might be capturing impulsivity and not smoking. This is further supported by the fact that when we used other instruments of smoking behaviour (e.g. lifetime smoking and smoking heaviness), we did not see any evidence for an effect, and Steiger filtering suggested that many of the SNPs for smoking initiation were not valid.

Self-harm is the result of complex interactions of personality factors, including impulsivity, social factors and mental state.^46^ It is well established that impulsivity is an important risk factor for suicide attempts.^47^ It has been shown that impulsivity increases with exposure to nicotine, returns to a normal levels with abstinence and increases with re-challenge after abstinence.^48,49^ The association with suicide attempts is not found in former smokers, and this therefore supports the link between smoking and impulsivity.^7,11,15,50^ This interaction between impulsivity, smoking initiation and suicide attempts is complex and further research is required. For example, portioning the biological (response to nicotine) and the behavioural (personality factors and risk-taking) components of the smoking initiation instrument will aid future interpretation of Mendelian randomisation analyses using smoking initiation instruments.

Recent theoretical models of suicide (i.e. integrated motivational volitional model^51^) all fit with the ‘ideation to action’ framework, which posits that the development of suicidal ideation and progression from ideation to attempts are distinct processes with separate risk factors and explanations.^52^ Clearly, this has important implications in clinical practice and risk management. Few studies have examined smoking within an ‘ideation to action’ framework. A cross-sectional study found suicide attempters were more likely than ideators to be current smokers.^53^ However, in the National Comorbidity Survey, early-onset nicotine dependence was prospectively associated with suicide plans, but not attempts, among those with ideation.^10^ Our single SNP analysis would support the idea of suicidal ideation and attempts being separate processes, differentially affected by smoking heaviness, and this differentiation is an important area that requires further research.

### Strengths and limitations

This study has many strengths, as to our knowledge, it is the first to use the method of Mendelian randomisation to explore the association between smoking and suicide attempts and ideation. We triangulated across multiple methods, multiple smoking behaviours and multiple suicidal behaviours to improve causal inference. However, the power of these analyses was limited by sample size. For example, we used a split half in the Mendelian randomisation of lifetime smoking, reducing power, which could explain why we saw no clear evidence for an effect. Furthermore, the single SNP analysis, designed to test the effect of smoking heaviness on suicidal ideation and attempts, included small numbers of those experiencing suicidal thoughts and suicide attempts, and was therefore underpowered. However, if anything, the trend of association was in the opposite direction to what was hypothesised. It should also be noted that the sample includes only non-fatal suicide attempts, and therefore our definition of suicide attempts is narrow and the results do not necessarily generalise to those who completed suicide. We were also limited by the UK Biobank questions asked for suicidal ideation and attempt. Suicide attempt questions refer to lifetime attempts but suicidal ideation questions refer to symptoms. Another possible limitation is bias from reverse causation. As attempts were unsuccessful, a pathway from attempts to smoking initiation is possible. Furthermore, Steiger filtering estimated that over half of the smoking initiation genetic instruments explained more variance in suicide attempts than smoking initiation. As discussed, this could be because of the instruments capturing underlying impulsivity and risk-taking.

In conclusion, this was the first Mendelian randomisation study to explore the effect of smoking on suicidal ideation and attempts. Our results suggest that, despite observed associations, when we triangulated across multiple Mendelian randomisation methods, there was no clear evidence for a causal effect of smoking behaviour. This supports recent evidence suggesting that suicidal ideation and suicide attempts are different processes. Furthermore, this work has highlighted the complexity of unpicking the behavioural from the biological components of smoking behaviours.

## Data Availability

For the summary-level Mendelian randomisation, we used publicly available summary statistics. For smoking initiation, we used summary data from the GWAS and Sequencing Consortium of Alcohol and Nicotine use GWAS.^27^ For suicide attempts, summary statistics from a GWAS conducted in the UK Biobank were used.^28^ All other analyses were conducted with data from the UK Biobank. This data is available upon request to approved researchers. The current project was conducted with data from UK Biobank, application number 9142.

## Supplementary material

For supplementary material accompanying this paper visit https://doi.org/10.1192/bjp.2020.68. click here to view supplementary material
