# Supplementary material for: Examining the effect of smoking on suicidal ideation and attempts: triangulation of epidemiological approaches
Source: Br J Psychiatry. 2020 Apr 15;217(6):701–7. doi: 10.1192/bjp.2020.68 (PMC7705667; doi:10.1192/bjp.2020.68)
Supplement: Supplementary file 1 [file S0007125020000689sup001.docx]

**Supplementary Materials**

**Contents**

| Table S1. Rucker’s Q Tests of heterogeneity in the SNP effects | Page 2 |
| --- | --- |
| Table S2. MR Egger intercept test of directional pleiotropy | Page 3 |
| Table S3. Summary level MR results after Steiger filtering | Page 4 |
| Table S4. Association of lifetime smoking score and polygenic risk score on the baseline confounders | Page 5 |
| Table S5. Baseline characteristics of 335,918 participants in the UK Biobank according to smoking status and number of rs1051730 effect alleles | Page 6 |
| Table S6: Mendelian randomisation analyses using summary level data of smoking initiation and risk-taking. | Page 7 |
| Table S7. Mendelian randomisation analyses using summary level data of smoking initiation on suicide attempts with smoking initiation SNPs restricted to independence at 10000kb and r2<0.001. | Page 8 |
|  |  |
|  |  |

**Table S1. Rucker’s Q Tests of heterogeneity in the SNP effects**

| **Exposure** | **Outcome** | **Method** | **Q** | **df** | **P-value** |
| --- | --- | --- | --- | --- | --- |
| Smoking initiation (378 SNPs) | Suicide attempts | IVW | 325.35 | 320 | 0.41 |
|  |  | MR Egger | 324.87 | 319 | 0.40 |
|  |  | Q’ | 0.48 | 1 | 0.50 |
| Smoking initiation (206 SNPs) | Suicide attempts | IVW | 182.76 | 179 | 0.41 |
|  |  | MR Egger | 182.26 | 178 | 0.40 |
|  |  | Q’ | 0.49 | 1 | 0.48 |
| Smoking initiation | Risk-taking | IVW | 1093.70 | 346 | <0.001 |
|  |  | MR Egger | 1043.79 | 345 | <0.001 |
|  |  | Q’ | 49.91 | 1 | <0.001 |
| Risk-taking | Smoking initiation | IVW | 192.75 | 100 | <0.001 |
|  |  | MR Egger | 191.28 | 99 | <0.001 |
|  |  | Q’ | 1.48 | 1 | 0.22 |

**Table S2. MR Egger intercept test of directional pleiotropy**

| **Exposure** | **Outcome** | **Intercept (95% CI)** | **P-value** |
| --- | --- | --- | --- |
| Smoking initiation (378 SNPs) | Suicide attempts | 0.003 (-0.006, 0.013) | 0.494 |
| Smoking initiation (206 SNPs) | Suicide attempts | 0.005 (-0.009, 0.019) | 0.489 |
| Smoking initiation | Risk-taking | 0.002 (0.001, 0.004) | <0.001 |
| Risk-taking | Smoking initiation | -0.002 (-0.005, -0.002) | 0.384 |

**Table S3. Summary level MR results after Steiger filtering**

| **Exposure** | **Outcome** | **N SNP** |
| --- | --- | --- |
| **Smoking initiation (378 SNPs)** | **Suicide attempt** | 153/367 (42%) |
| **Smoking initiation (206 SNPs)** | **Suicide attempt** | 78/180 (43%) |
| **Smoking initiation** | **Risk-taking** | 304/347 (88%) |
| **Risk-taking** | **Smoking initiation** | 118/123 (96%) |

**Table S4. Association of lifetime smoking score and polygenic risk score on the baseline confounder**

|  | **Observed association with lifetime smoking** | | **Genetic association with lifetime smoking polygenic score** | |
| --- | --- | --- | --- | --- |
|  | **Test statistic** | **p-value** | **Test statistic** | **p-value** |
| **Sex** | t = -29.52 (1, 154080) | P<0.001 | t = 0.96 (1, 164010) | P=0.338 |
| **Age** | $\beta$ = 0.002 (SE = 0.0002) | P<0.001 | $\beta$ = -0.0006 (SE = 0.0003) | P=0.035 |
| **Socio-economic position** | $\beta$ = 0.046 (SE = 0.0006) | P<0.001 | $\beta$ = 0.005 (SE = 0.0008) | P<0.001 |
| **Educational attainment** | $\beta$ = -0.164 (SE = 0.002) | P<0.001 | $\beta$ = -0.028 (SE = 0.004) | P<0.001 |
| **Alcohol consumption** | $\beta$ = -0.004 (SE = 0.001) | P=0.011 | $\beta$ = -0.005 (SE = 0.002) | P=0.017 |

# **Table S5: Baseline characteristics of 335,918 participants in the UK Biobank according to smoking status and number of rs1051730 effect alleles**

|  |  | Never smokers | | | |  | |  | |  | | Ever smokers | | | |  | |  | |
| --- | --- | --- | --- | --- | --- | --- | --- | --- | --- | --- | --- | --- | --- | --- | --- | --- | --- | --- | --- |
|  |  | **0** | | **1** | | **2** | | **p-value** | |  | | **0** | | **1** | | **2** | | **p-value** | |
| Study sample (n) |  | 82,130 | | 81,580 | | 20,334 | |  | |  | | 68,562 | | 66,953 | | 16,307 | |  | |
| Cigarettes per day (mean) | |  |  | |  | |  | |  | | 14.88 | | 15.89 | | 16.72 | | <0.001 | |  |
| Missing (n) |  |  | |  | |  | |  | |  | | 3807 | | 3371 | | 739 | |  | |
| Smoking status (%)^a^ |  |  | |  | |  | |  | |  | |  | |  | |  | |  | |
| Current |  |  | |  | |  | |  | |  | | 22 | | 22 | | 22 | |  | |
| Former |  |  | |  | |  | |  | |  | | 78 | | 78 | | 78 | | 0.057 | |
| Men (%) |  | 42 | | 41 | | 41 | | 0.121 | |  | | 52 | | 52 | | 52 | | 0.539 | |
| Age (mean) |  | 56.1 | | 56.2 | | 56.2 | | 0.092 | |  | | 57.8 | | 57.7 | | 57.6 | | 0.002 | |
| Education (%) | |  |  | |  | |  | |  | |  | |  | |  | |  | |  |
| Primary |  | 14 | | 14 | | 14 | |  | |  | | 21 | | 21 | | 20 | |  | |
| Secondary |  | 51 | | 50 | | 50 | |  | |  | | 51 | | 52 | | 52 | |  | |
| Tertiary |  | 36 | | 36 | | 36 | | 0.776 | |  | | 27 | | 27 | | 28 | | 0.020 | |
| Missing (n) |  | 683 | | 704 | | 202 | |  | |  | | 634 | | 685 | | 133 | |  | |
| Alcohol intake (%) |  |  | |  | |  | |  | |  | |  | |  | |  | |  | |
| Never |  | 7 | | 7 | | 7 | |  | |  | | 5 | | 6 | | 6 | |  | |
| Special occasions |  | 11 | | 12 | | 11 | |  | |  | | 9 | | 9 | | 9 | |  | |
| Monthly |  | 12 | | 13 | | 12 | |  | |  | | 9 | | 10 | | 10 | |  | |
| Weekly |  | 53 | | 52 | | 53 | |  | |  | | 49 | | 48 | | 47 | |  | |
| Daily/almost daily |  | 16 | | 16 | | 16 | | 0.161 | |  | | 28 | | 28 | | 28 | | 0.074 | |
| Missing (n) |  | 33 | | 47 | | 16 | |  | |  | | 47 | | 55 | | 13 | |  | |
| Suicidal Ideation (%) |  | 3.6 | | 3.6 | | 3.1 | | 0.068 | |  | | 4.9 | | 4.7 | | 5.3 | | 0.242 | |
| Missing (N) |  | 53,810 | | 53,424 | | 13,250 | |  | |  | | 47,941 | | 46,651 | | 11,352 | |  | |
| Attempted suicide (%) |  | 1.6 | | 1.6 | | 1.4 | | 0.596 | |  | | 3.2 | | 3.0 | | 2.6 | | 0.086 | |
| Missing (N) |  | 53,719 | | 53,327 | | 13,237 | |  | |  | | 47,843 | | 46,574 | | 11,341 | |  | |

**Table S6. Mendelian randomisation analyses using summary level data of smoking initiation and risk-taking.**

| **Exposure** | **Method** | **N SNP** | **Beta (95% CI)** | **P-value** |
| --- | --- | --- | --- | --- |
| **Smoking initiation** | Inverse-Variance Weighted | 347 | 0.284 (0.228, 0.341) | <0.001 |
|  | Weighted Median | 347 | 0.205 (0.149, 0.261) | <0.001 |
|  | Weighted Mode | 347 | 0.210 (0.061, 0.360) | 0.006 |
|  | MR RAPS | 347 | 0.303 (0.240, 0.365) | <0.001 |
| **Risk-taking** | Inverse-Variance Weighted | 101 | 0.152 (0.081, 0.225) | <0.001 |
|  | Weighted Median | 101 | 0.172 (0.092, 0.252) | <0.001 |
|  | Weighted Mode | 101 | 0.192 (0.017, 0.366) | 0.03 |
|  | MR RAPS | 101 | 0.176 (0.103, 0.248) | <0.001 |

**Table S7. Mendelian randomisation analyses using summary level data of smoking initiation on suicide attempts with smoking initiation SNPs restricted to independence at 10000kb and r2<0.001.**

| **Method** | **N SNP** | **OR (95% CI)** | **P-value** |
| --- | --- | --- | --- |
| Inverse-Variance Weighted | 180 | 2.84 (1.62, 5.00) | <0.001 |
| Weighted Median | 180 | 2.68 (1.14, 6.30) | 0.02 |
| Weighted Mode | 180 | 3.24 (0.48, 22.00) | 0.23 |
| MR RAPS | 180 | 3.30 (1.69, 6.43) | <0.001 |
